# Correlation Between the Peak of Skin Thickness Progression Rate and Onset of Cardiopulmonary Involvement in Thai Systemic Sclerosis Patients

**DOI:** 10.3390/jcm14072281

**Published:** 2025-03-27

**Authors:** Piyanart Rujirawinitchai, Chingching Foocharoen, Ajanee Mahakkanukrauh, Siraphop Suwannaroj, Patnarin Pongkulkiat, Tippawan Onchan

**Affiliations:** Division of Rheumatology, Department of Medicine, Faculty of Medicine, Khon Kaen University, Khon Kaen 40002, Thailand; pongpang_r@hotmail.com (P.R.);

**Keywords:** systemic sclerosis, scleroderma, interstitial lung disease, pulmonary hypertension, pulmonary arterial hypertension, modified Rodnan skin score

## Abstract

**Background/Objectives:** Rapid skin thickness progression assessed using the modified Rodnan skin score (mRSS) is associated with poor outcomes in systemic sclerosis (SSc). However, the correlation between patterns of skin thickness and the onset of internal organ involvement remains unclear. This study aimed to determine the correlation between peak skin thickness progression rate (pSTPR) and the onset of internal organ involvement, particularly interstitial lung disease (ILD) and pulmonary arterial hypertension (PAH) among Thai SSc patients. **Method:** A prospective cohort study with retrospective analysis was conducted on adult SSc patients who experienced the onset of their first non-Raynaud phenomenon symptoms between January 2013 and December 2020 and had at least a 2-year follow-up. Patients with an overlap syndrome were excluded from this study. The pSTPR was calculated by dividing the peak of the mRSS by the duration of disease at the peak of the mRSS. **Result:** A total of 509 patients were included in this study. The majority of cases were female (351; 69.0%) and comprised diffuse cutaneous SSc subsets (353 cases; 69.4%). The respective mean age and median pSTPR was 48.2 ± 11.6 years and 1.63 points/year (interquartile range 0.5–4.4). The respective median durations of disease at the onset of significant ILD (>20% extent), ILD, and mean duration of disease at the onset of PAH were 3.4 (Q1–Q3 1.4–7.7), 5.4 (Q1–Q3 2.4–9.2), and 8.0 ± 4.9 years. pSTPR was negatively correlated with disease duration at the onset of significant ILD (Rho −0.509, *p* < 0.001), ILD (Rho −0.480, *p* < 0.001), PAH (Rho −0.372, *p* = 0.03), and disease duration from onset to death (Rho −0.367, *p* = 0.03) after adjusting for age, sex, and anti-topoisomerase I. **Conclusion:** SSc patients with a high skin thickness progression rate reaching the maximum point of mRSS were at risk of developing early ILD, PAH, and death. The pSTPR may be used to assess individuals at risk of experiencing early onset cardiopulmonary involvement in SSc.

## 1. Introduction

Systemic sclerosis (SSc) is a complex autoimmune connective tissue disease that causes obliterative vasculopathy and inflammation, which subsequently leads to fibrosis of the skin and internal organs. SSc is classified into two major groups: limited cutaneous systemic sclerosis (lcSSc) and diffused cutaneous systemic sclerosis (dcSSc) [1]. Skin thickness is the major characteristic presentation in SSc patients, evaluated using the modified Rodnan skin score (mRSS), which is simple, valid, and reliable [2,3,4]. The mRSS assesses the skin in 17 areas: the face, chest, abdomen, arms, forearms, hands, fingers, thighs, legs, and feet [5]. The scores are described in 0–3 scores in each area.

Assessment of the severity and extent of skin thickness is important as it is a surrogate marker of disease activity, severity, and prognosis. According to the findings, a high skin score is associated with poor self-reported physical health status [6]. Some studies found that a greater extent and severity of initial skin involvement were predictive of renal and cardiac involvement and decreased survival. A cohort study by Domsic et al. [7] studied 826 early dcSSc cases and reported that in patients with a rapid skin thickness progression rate, there were dependent risk factors of mortality and renal crisis in 2011. A similar study in 2019 examined 1021 dcSSc cases and found that progressive skin fibrosis within 1 year was associated with a decline in lung function and a lower survival rate [8].

The prevalence of SSc in Thailand was 24.4 per 100,000 persons, according to the National Health Security Office of Thailand [9]. The majority of cases in Thailand are dcSSc [10], which has a worse prognosis [1] and more severe internal organ complications and poor quality of life compared with lcSSc [11]. There was a high incidence of interstitial lung disease (ILD), and the main cause of death in Thai patients with early SSc is cardiac involvement [12,13]. Wangkaew et al. [14] showed that skin change determined by skin thickness progression rate (STPR) at the baseline visit was a marker for cardiac and ILD complications among Thais with SSc. However, the authors did not evaluate the correlation between the onset of cardiopulmonary complications and the skin thickness progression rate, which could be useful information in daily practice. Foocharoen et al. [15] revealed that the most common skin pattern in SSc patients with SSc was slow progression to peak, followed by slow regression. The average peak skin score assessed by mRSS and the average duration of disease at peak skin score was 19.8 points and 20.3 months, respectively. However, the authors found no correlation between skin thickness patterns and internal organ involvement.

Data from previous studies indicate that mRSS may be correlated with internal organ complications. To date, there has been no study on the correlation between the rate of skin thickness progression to peak mRSS and the onset of internal organ complications, and awareness of the progression of skin thickness is important in clinical practice. We hypothesized that a high peak skin thickness progression rate (pSTPR) may be associated with the short onset of ILD and pulmonary arterial hypertension (PAH) in patients with SSc patients. Hence, our objective was to evaluate the correlation between pSTPR and the onset of significant ILD and PAH.

## 2. Methods

### 2.1. Study Design and Population

A prospective cohort study with retrospective analysis was conducted among adult SSc patients who experienced the onset of their first non-Raynaud phenomenon symptoms between January 2013 and December 2020 and had at least a 2-year follow-up after onset. Eligible patients were enrolled from the scleroderma clinic, Srinagarind Hospital, Khon Kaen University, Khon Kaen, Thailand. All patients provided written informed consent before enrolment in the cohort study. The diagnosis of SSc was based on the 2013 ACR/EULAR Classification Criteria for Scleroderma [16]. LeRoy classification was used for classifying dcSSc and lcSSc [1]. Patients with overlap syndrome, previous history of PAH or ILD before the diagnosis of SSc, unclear onset of ILD or PAH, and having a peak of skin thickness at onset or on the first visit were excluded.

Demographic data (age, sex, occupation, and comorbidities). SSc-related data (clinical symptoms and laboratory tests, including autoantibodies) were extracted from the database of the Scleroderma Research Group. The mRSS assessment was previously validated for inter- and intra-observer variability and was conducted by trained rheumatologists [17].

### 2.2. Operation Definition

The start date or date of disease onset was the date of the first non-Raynaud’s phenomenon symptom of SSc, and the end date was the end date of the study (date of the last follow-up). The pSTPR was calculated by dividing the peak of the mRSS by the duration of disease at the peak of the mRSS, as shown below.

$$\mathrm{pTPR}\;\left(\mathrm{points}/\mathrm{year}\right)=\frac{\mathrm{Peak}\;\mathrm{of}\;\mathrm{mRSS}}{\mathrm{Duration}\;\mathrm{of}\;\mathrm{disease}\;\mathrm{at}\;\mathrm{peak}\;\mathrm{of}\;\mathrm{mRSS}}$$

The peak of mRSS was the highest when it was sustained at least twice on consecutive follow-up visits. Disease duration at the peak of mRSS was calculated by the interval time between the onset of the first non-Raynaud’s phenomenon symptoms and the date of having a peak of mRSS. PAH was defined when the mean pulmonary artery pressure was >20 mmHg with PCWP ≤ 15 mmHg with PVR ≥ 2 WU by right heart catheterization [18]. The date at onset of PAH was the date of PAH diagnosis. Disease duration at the onset of PAH was calculated by subtracting the date of onset of PAH from the date of disease onset. ILD was considered present when interstitial fibrosis was detected by high-resolution computed tomography (HRCT), and significant ILD was detected when HRCT detected ILD involving more than 20% of the total lung volume. The date of ILD onset was the date of diagnosis. Disease duration at the onset of ILD was calculated by subtracting the date of onset of ILD from the date of disease onset.

A digital ulcer was defined as a painful denuded area with well-demarcated borders located on the volar aspect of the finger [12]. Hand deformity was characterized by flexion contractures of the finger joints, resembling claw deformities [19]. Esophageal involvement was identified when any symptoms related to the esophagus in SSc manifested, such as heartburn, esophageal dysphagia, or reflux symptoms [12]. Stomach involvement was characterized by symptoms such as vomiting or early satiety [12]. Intestinal involvement was identified by symptoms including malabsorption, diarrhea, constipation, bloating, pseudo-intestinal obstruction, and/or ileus [12]. Myocardial involvement was characterized when the left ventricular ejection fraction was equal to or less than 50%. The renal crisis was signified by (a) the sudden onset of hypertension, a rapid, progressive (b) increase in serum creatinine, and/or (c) microangiopathic hemolytic anemia [12]. Weight loss is indicated as the unintended loss of more than 5 percent of one’s usual body weight over a period of 6 to 12 months [12]. Anemia was defined as hemoglobin levels below 13.0 g/dL in males and below 12.0 g/dL in females [12].

Patients were censored if they were lost to follow-up or remained alive at the end of the study period. Loss-to-follow-up status was obtained from the government office, and the information was reviewed, with the cause of death ascertained by a physician.

### 2.3. Statistical Analysis

The sample size was calculated using the formula N = [(Z_α_ + Z_β_)/C]^2^ + 3, where Z_α_ and Z_β_ are the standard normal deviations for α and β, respectively. C was calculated using the formula C = 0.5 × ln[(1 + r)/(1 − r)], where r is the correlation coefficient. With 80% power (β) at a significance level (α) of 0.05 and a correlation coefficient (r) of 0.429, according to a previous study by Hikmat et al. [20], the authors revealed a correlation between ILD morphology scores based on HRCT and skin fibrosis degree based on mRSS with r = 0.429; the sample size was determined to be at least 42 cases. However, we included all eligible SSc patients according to the method of the study in the analysis.

Clinical characteristics were categorized as dichotomous, polytomous, or continuous variables. The distribution of the continuous data was evaluated using the Shapiro–Wilk test. The continuous variables were presented as mean and standard deviation (SD) if the data followed a normal distribution and as median and interquartile range (IQR/Q1–Q3) if the data were skewed. Categorical variables are expressed as proportions or percentages. The correlation between pSTPR and the onset of significant ILD, ILD, and PAH was analyzed using Spearman Rank correlation and adjusted for age at onset, sex, and antibody, particularly anti-topoisomerase I. Statistical significance was set at 0.05. All data analyses were conducted using STATA version 16.0 (StataCorp., College Station, TX, USA).

## 3. Result

A total of 803 SSc patients from the database were included, of which 294 cases were excluded from this study. A total of 509 patients were analyzed, with 6081 person-years. The majority were female (351 cases; 69%), and the mean age was 48.2 ± 11.6 years. The predominant subset of SSc cases was dcSSc (353 cases, 69.4%). Clinical characteristics on the last follow-up were categorized into general, skin and musculoskeletal, gastrointestinal, respiratory, and other symptoms. The patients’ demographic data, laboratory results, and overall complications are shown in Table 1.

The respective median durations from disease onset to significant ILD and ILD diagnosis were 3.4 (Q1–Q3 1.4–7.7) and 5.4 (Q1–Q3 2.4–9.2). The mean duration from disease onset to PAH diagnosis was 8.0 ± 4.9 years. The mean peak mRSS was 13.2 ± 10.5 points, with a duration of disease on the date of peak mRSS detection of 8.0 ± 6.2 years. The median pSTPR was 1.6 points/year (IQR 0.5–4.4). The mean duration of disease to the date of death was 10.6 ± 5.3 years.

According to the objectives of the study, pSTPR exhibited a negative correlation with disease duration at the onset of ILD (Rho −0.707, *p* < 0.001), disease duration at the onset of significant ILD (Rho −0.677, *p* < 0.001), and disease duration at the onset of PAH (Rho −0.552, *p* < 0.001). In addition, pSTPR was negatively correlated with disease duration from onset to death (Rho −0.581, *p* < 0.001). After adjusting for age at onset, sex, and anti-topoisomerase I, the correlation remained significant with a Rho of −0.325 for disease duration at the onset of significant ILD (*p* < 0.001), −0.318 for ILD (*p* < 0.001), −0.316 for PAH (*p* < 0.001), and −0.402 for disease duration from onset to death (*p* < 0.001). After adding anti-topoisomerase I as an adjusted variable, the correlation between pSTPR and disease duration at the onset of significant ILD, ILD, PAH, and death remained significant in the same direction as no antibody adjustment (Table 2).

## 4. Discussion

We conducted a prospective cohort study of Thai SSc patients with a retrospective analysis of the database from the Scleroderma registry. There was a significant correlation between the pSTPR and the onset of cardiopulmonary complications. Although there is evidence that high levels of anti-topoisomerase I are correlated with a shorter onset of cardiopulmonary involvement [21], our analysis showed a negative correlation between pSTPR and the duration of disease at the onset of significant ILD, ILD, PAH, and time to death, after adjusting for age at onset, sex, and specific autoantibody (anti-topoisomerase I), which is related to worse prognosis among SSc patients. Our findings indicated a higher pSTPR, shorter onset of cardiopulmonary complications, particularly ILD and PAH, and shorter survival. Hence, the rapid progression of skin fibrosis may serve as a predictor of early cardiopulmonary involvement in SSc patients and the potential utility of pSTPR as a prognostic marker for early cardiopulmonary involvement and mortality among SSc patients. However, we did not include patients with a previous history of PAH or ILD before the diagnosis of SSc or those with an unclear onset of ILD or PAH because they were not within the scope of our study to define the correlation between pSTPR and the onset of internal organ involvement.

The substantial association between skin and pulmonary fibrosis, particularly in the context of ILD in SSc patients, has been underscored by several clinical observations. It is postulated that skin thickness and SSc-ILD share a synchronous progression, typically manifesting within the initial three years of the disease’s natural course [22]. Consequently, vigilant monitoring of patients during this critical period is imperative to detect early signs and symptoms indicative of subsequent severe organ complications. Attributed to similarities in pathophysiological mechanisms underlying both skin and lung involvement in SSc, early stages are marked by infiltration of inflammatory cells, succeeded by proliferation accompanied by collagen fiber degeneration in later stages [22]. The immunopathogenic mechanisms driving both skin fibrosis, ILD, and PH in SSc involve the same several key cytokines, chemokines, and cellular pathways, including transforming growth factor beta, interleukin-6, and interleukin-4. Endothelial dysfunction triggers vascular damage, immune activation, and the production of endothelin-1 and platelet-derived growth factors. Myofibroblast activation, mediated by TGF-B signaling, results in excessive extracellular matrix production, contributing to both skin and lung fibrosis [23,24]. It suggests that skin and lung fibrosis in SSc has a similar abnormality of the immune system as its background [25]. Clinical responses to therapeutic interventions further corroborate this relationship, revealing favorable outcomes in both skin and lung fibrosis with immunosuppressants, including mycophenolate mofetil, tocilizumab, and B cell-targeting therapies [26,27,28]. Moreover, empirical evidence from previous investigations, such as the study conducted by Matsuda et al. [25] in 2019 in Japan, demonstrates a notable correlation between skin scores and pulmonary function, particularly pronounced in patients with shorter disease durations (*p* < 0.05). Similarly, findings from Cottrell et al. [29] in 2014, based on the United States population, elucidate that patients with elevated skin fibrosis scores may progress to moderate to severe restrictive pulmonary involvement (*p* < 0.001) [29]. These findings align with our own, indicating a significant correlation between high pSTPR and the rapid onset of both significant ILD and ILD. Collectively, these insights underscore the potential clinical utility of monitoring skin fibrosis progression as an indicator of early cardiopulmonary involvement in SSc patients.

PAH is frequent and may be severe in patients with SSc. The major issue is that there is not one single pulmonary hypertension group in the World Symposium on Pulmonary Hypertension (WSPH) classification and one single mechanism at play in SSc. Several studies have revealed controversy regarding the relationship between skin thickness progression and pulmonary hypertension. Previous reports have presented conflicting findings regarding the association between worsened skin thickness and the occurrence of pulmonary hypertension, utilizing various definitions of pulmonary hypertension [7,8,15,30]. Some similar studies, including those by Shand et al. [31], have found no significant relationship between the total skin thickness score or its progression and the development of pulmonary or cardiac involvement. Conversely, other studies have suggested a potential link between skin thickness progression and multiorgan involvement. For instance, dcSSc patients exhibiting intermediate to rapid skin thickness progression at initial evaluation were shown to be at a significantly higher risk of developing both renal and cardiac complications [14], as observed in our study, where a higher pSTPR was associated with a shorter duration until the onset of PAH.

The relationship between skin thickness and mortality in SSc was clearly observed and is supported by our observations. We found that the higher the pSTPR, the shorter the survival time. Previous reports also revealed that patients with high STPR had a significantly increased risk of mortality due to renal crisis and heart disease [7,32]. The summarized data on the correlation between skin thickness progression rate and clinical outcomes of SSc are presented in Table 3. The discrepancies observed across studies can be attributed to variations in genetics, patient characteristics, sample sizes, definitions of organ involvement, and study duration. Indeed, the complexity of SSc pathogenesis and its diverse clinical manifestations require further investigation through larger-scale and more comprehensive studies.

The findings of this study offer valuable insights into the association between peak skin thickness progression rate (pSTPR) and cardiopulmonary involvement in Thai patients with SSc, a condition frequently associated with significant morbidity and mortality. Understanding the factors that influence disease progression and organ involvement is paramount for enhancing patient care and outcomes. The potential for predicting early problems in patients is enormous when a useful and accessible surrogate marker or tool, such as the pSTPR, is made available. Clinical results in this patient population may be improved by the early detection and monitoring of changes in skin thickness, which may allow for prompt intervention and management of cardiopulmonary problems. For example, in patients with high pSTPR, early referral for HRCT scans may be necessary rather than following conventional timetables, which could postpone the diagnosis and treatment of lung involvement.

The strength of this study lies in its inclusion of a large number of participants. Currently, there is a paucity of research exploring the relationship between skin thickness progression rate and internal organ involvement in patients with SSc, adjusting for age, sex, and specific autoantibody (anti-topoisomerase I), which is related to the poor prognosis of SSc. This study contributes significantly to expanding our understanding of this relationship and underscores the importance of further research in this area.

However, our study had some limitations, including (a) the possibility of missing data inherent to natural observational studies and (b) some confounders, such as smoking status, comorbidity, and concomitant medication, were not included in the analysis. This approach may have introduced selection bias and limited data availability, (c) a variation in the modified Rodnan Skin Score (mRSS), which is a subjective measure. However, we attempted to address this limitation by utilizing the closest two stable measurements of peak mRSS to minimize aberrations and use inter-rater or intra-rater reliability among physicians when evaluating the mRSS, (d) our study cohort comprised solely Thai patients and single center, so the generalizability of our findings to other ethnic populations may be limited. The majority of our study population has dcSSc, which is associated with more rapid disease progression, a lower 10-year survival rate, and a higher risk of early mortality due to ILD cardiac involvement. On the other hand, in the US and Europe, the lcSSc is more prevalent and characterized by slower disease progression. Therefore, caution should be exercised when extrapolating these results to other demographic groups. Further prospective studies involving larger and more diverse cohorts are required to validate our findings. Additionally, such studies could provide a deeper understanding of the underlying mechanisms driving the observed correlation between pSTPR and cardiopulmonary involvement in SSc. These findings may help in early assessment and prognostic evaluation, followed by modifications to treatment plans or interventions for better long-term results.

## 5. Conclusions

Negative correlations were observed between pSTPR and disease duration at the onset of cardiopulmonary involvement in SSc, including ILD and PAH, as well as the survival time. These findings suggest that pSTPR monitoring may serve as a valuable predictor of cardiopulmonary involvement in patients with SSc and as a marker of poor prognosis. Early identification and monitoring of changes in the pSTPR could facilitate timely intervention and management, ultimately leading to improved clinical outcomes in this patient population.

## Figures and Tables

**Table 1:** Patient demographics and clinical on the last follow-up.

Data	N = 509
Demographics	
Age at onset (years), mean ± SD	48.2 ± 11.6
Sex: female, n (%)	351 (69)
dcSSc subset, n (%)	353 (69.4)
Duration of disease (years), mean ± SD	11.9 ± 5.9
Skin & Musculoskeletal symptom	
Peak of mRSS (points), median (Q1–Q3)	11 (4–19)
Raynaud’s phenomenon, n (%)	251 of 504 (49.8)
Digital ulcer, n (%)	121 0f 504 (24.0)
Digital gangrene, n (%)	8 of 504 (1.6)
Telangiectasia, n (%)	202 0f 504 (40.0)
Calcinosis cutis, n (%)	44 0f 504 (8.7)
Salt and pepper appearance, n (%)	162 of 504 (32.1)
Edematous skin, n (%)	21 of 504 (4.2)
Tendon fiction rub, n (%)	56 of 504 (11.1)
Hand deformity, n (%)	199 of 504 (39.5)
Synovitis, n (%)	24 of 504 (4.8)
Gastrointestinal symptoms	
Esophageal involvement, n (%)	214 (42.0)
Stomach involvement, n (%)	79 of 504 (15.7)
Intestinal involvement, n (%)	81 (15.9)
General symptoms	
WHO functional class	
I, n (%)	153 of 363 (42.2)
II, n (%)	159 of 363 (43.8)
III, n (%)	44 of 363 (12.1)
IV, n (%)	7 of 363 (1.9)
Constitutional symptoms, n (%)	41 of 504 (8.1)
Weight loss, n (%)	75 of 504 (14.9)
Laboratory data	
Hemoglobin (g/dL), mean ± SD	12.3 ± 5.3
CRP (mg/dL), median (Q1–Q3)	3.1 (1.3–8.9)
Creatinine (mg/dL), median (Q1–Q3)	0.8 (0.6–1.0)
Albumin (g/dL), mean ± SD	4.0 ± 0.7
CK (IU/L), median (Q1–Q3)	93 (64–140.5)
NT-ProBNP (pg/mL), median (Q1–Q3)	146 (47–1058)
hs-cTnT (ng/L), median (Q1–Q3)	14.1 (7.4–31.2)
Anti-topoisomerase I positive, n (%)	239 of 314 (76.1)
Complications	
ILD	282 (55.4)
Disease duration at onset of ILD (years), median (Q1–Q3)	5.4 (2.4–9.2)
Significant ILD	54 (10.6)
Duration of disease at onset of significant ILD (years), median (Q1–Q3)	3.4 (1.4–7.7)
PAH	82 of 507 (16.2)
Disease duration at onset of PAH (years), mean ± SD	8.0 ± 4.9
Renal crisis, n (%)	1 of 417 (0.2)
Death, n (%)	153 (30.1)
Age at death (years), mean ± SD	60.5 ± 11.7
Disease duration at death (years), mean ± SD	10.6 ± 5.3
Disease duration at peak mRSS (years), median (Q1–Q3)	6.7 (3.4–11.0)
pSTPR (points/years), median (Q1–Q3)	1.6 (0.5–4.4)

SD, standard deviation; dcSSc, diffuse cutaneous systemic sclerosis; IQR, interquartile range; mRSS, modified Rodnan skin score; WHO, World Health Organization; CRP, C-reactive protein; ILD, interstitial lung disease; PAH, pulmonary arterial hypertension; CK, creatine kinase; NT-ProBNP, N-terminal pro-brain natriuretic peptide; hs-cTnT, high-sensitivity cardiac troponin T; pSTPR, peak skin thickness rate.

**Table 2:** Correlation between pSTPR and onset of significant ILD, ILD, and PAH.

Data	No Adjustment		Adjustment for Age at Onset and Sex		Adjustment for Age at Onset, Sex, and Positive for Anti-Topoisomerase I	
	Rho	*p*-Value	Rho	*p*-Value	Rho	*p*-Value
Disease duration at onset of significant ILD	−0.677	<0.001 *	−0.325	<0.001 *	−0.509	<0.001 *
Disease duration at onset of ILD	−0.707	<0.001 *	−0.318	<0.001 *	−0.480	<0.001 *
Disease duration at onset of PAH	−0.552	<0.001 *	−0.316	<0.001 *	−0.372	0.03 *
Disease duration at onset to die	−0.581	<0.001 *	−0.402	<0.001 *	−0.367	0.01 *

* Statistically significant. ILD; interstitial lung disease, PAH; pulmonary arterial hypertension.

**Table 3:** Summarized data of the correlation between skin thickness progression rate and clinical outcomes of SSc.

Authors	Country	Year of Study	N	Study Design	Findings
Our study	Thailand	2013–2020	509	Cohort study	- Negative correlation between pSTPR and disease duration at onset of significant ILD, ILD, PAH, and death after adjustment for age at onset, sex, and anti-topoisomerase I with Rho of −0.509, −0.480, −0.372, and −0.367, respectively.
Wu et al. [8]	EUSTAR database	2009–2017	1021	Cohort study	- Progressive skin fibrosis within 1 year is associated with decline in FVC ≥ 10% (HR 1.79, 95% CI 1.20–2.65) and worse survival during follow-up. (all cause death (HR 2.58, 95% CI 1.31–5.09)
Domsic et al. [7]	USA	1980–2005	826	Cohort study	- Rapid STPR was an increased risk of mortality and develop renal crisis within 2 years of follow-up with OR 1.72; 95% CI 1.13–2.62; *p* = 0.01 and OR 2.05, 95% CI 1.10–3.85; *p* = 0.02, respectively.
Steen et al. [33]	USA	1972–1994	278	Cohort study	- Improvement in skin thickening of dcSSc is associated with improved survival. - No differences in the occurrence of severe organ involvement between two groups of improvement or no improvement of skin thickening during the 2 years follow-up
Shand et al. [31]	UK	1983–2001	225	Cohort study	- A high skin thickness score is associated with increased mortality (*p* = 0.003). - No relationship between the pattern of change in the mRSS and the frequency of internal organ involvement (lung, heart, kidney or skeletal muscle) over time (*p* = 0.005).
Perera et al. [32]	USA	1980–2001	212	Cohort study	- SSc cardiac disease was found significantly more often in patients with rapid versus slow STPR (*p* = 0.006) - ATA positive patients with a rapid STPR have reduced survival rates, primarily due to early and often fatal renal and cardiac involvement.
Matsuda et al. [25]	Japan	2011–2018	198	Cohort study	- Higher mRSS is related to a higher prevalence of interstitial lung disease (*p* < 0.05), restrictive impairment (*p* < 0.01), and diffusion impairment (*p* < 0.05) of the lung. - Negative correlates between mRSS and %FVC (*p* < 0.001) and diffusing capacity (*p* < 0.001) of the lung.
Wannarong et al. [30]	Thailand	2013–2016	118	Cohort study	- Higher mRSS over 1 year was significant associated with internal organ involvement.
Foocharoen et al. [15]	Thailand	2005–2006	117	Cohort study	- The most common skin pattern in Thai SSc was slow progression to peak then slow regression. - Telangiectasia at onset and contracture of joint(s) were predictive of continuous progressive skin thickness in the first 3 years with *p* = 0.001 and *p* = 0.042, respectively. - Skin thickness pattern does not correlate with SSc subsets and internal organ involvement.
Wangkaew et al. [14]	Thailand	2010–2017	104	Cohort study	- Rapid skin progression is associated with a higher incidence rate of LVEF < 50% and ILD complications than those of slow skin progression - Skin non-improvers had higher mortality than skin improvers.
Hikmat et al. [20]	Indonesia	2019–2020	42	Cohort study	- Positive correlation between skin fibrosis based on mRSS and morphological scores of ILD with R = 0.429.
Rohman et al. [34]	Indonesia	2021	23	Cross sectional study	- Significant relationship between mRSS and pulmonary fibrosis (r = 0.485, *p* = 0.019), pulmonary hypertension (r = 0.63, *p* = 0.001).

dcSSc, diffuse cutaneous systemic sclerosis; SSc, systemic sclerosis; mRSS, modified Rodnan skin score; FVC, forced vital capacity; STPR, skin thickness progression rate; LVEF, left ventricular ejection fraction; ATA: Anti-topoisomerase antibody.

## Data Availability

The datasets used and/or analyzed during the current study are available from the corresponding author upon reasonable request.
